# Wax, sex and the origin of species: Dual roles of insect cuticular hydrocarbons in adaptation and mating

**DOI:** 10.1002/bies.201500014

**Published:** 2015-05-19

**Authors:** Henry Chung, Sean B Carroll

**Affiliations:** Howard Hughes Medical Institute and Laboratory of Molecular Biology, University of WisconsinWI, USA

**Keywords:** chemical communication, cuticular hydrocarbons, desiccation, ecological adaptation, mating success, speciation

## Abstract

Evolutionary changes in traits that affect both ecological divergence and mating signals could lead to reproductive isolation and the formation of new species. Insect cuticular hydrocarbons (CHCs) are potential examples of such dual traits. They form a waxy layer on the cuticle of the insect to maintain water balance and prevent desiccation, while also acting as signaling molecules in mate recognition and chemical communication. Because the synthesis of these hydrocarbons in insect oenocytes occurs through a common biochemical pathway, natural or sexual selection on one role may affect the other. In this review, we explore how ecological divergence in insect CHCs can lead to divergence in mating signals and reproductive isolation. We suggest that the evolution of insect CHCs may be ripe models for understanding ecological speciation.

## Introduction

The processes of adaptation and speciation have been linked conceptually since Darwin, who suggested that adaptation to different environments leads to the evolution of new traits and new species [1]. In the past few decades, aided by advances in molecular and developmental genetics, biologists have elucidated the genetic mechanisms underlying the evolution of scores of animal traits [2]. However, how evolutionary changes in traits may lead to reproductive isolation and the formation of new species is less understood. Speciation requires the formation of reproductive barriers between populations. If changes in other traits are necessary to establish reproductive isolation, it has not been clear how adaptation and speciation are linked mechanistically.

The role of ecology in the speciation process has been receiving increasing attention of late [3,4]. It has been proposed that traits that have dual roles in ecological adaptation, and that mating success could generate reproductive barriers, even in non-allopatric conditions [5] (Box 1). Divergent selection acting on these traits could then produce non-random mating in populations and lead to reproductive isolation. Traits that contribute to both ecological divergence and non-random mating in populations were once thought to be rare and unusual, but many candidates have now been identified [5].

**Box 1 Dual Traits, multiple-effect traits and so-called “magic traits”**

Biologists largely agree that ecological factors play a major role in speciation. However, the mechanisms underlying the progression from ecological selection to reproductive isolation have not been well defined until recently [77]. One potential mode of ecological speciation is manifest when single traits that are under divergent ecological selection also cause non-random mating [5]. These traits were dubbed “magic traits” because they were once hypothesized to be rare and theoretically difficult to envisage occurring in nature, especially when speciation is non-allopatric [78].

However, a recent survey suggested that these traits are not as rare as once thought, and several candidate examples of such traits across multiple taxa have been identified [5]. These traits have also been called “multiple-effect traits” – to distinguish between traits and their effects – and it has been suggested that the term “magic” is misleading because it implies that these traits automatically cause speciation or could somehow circumvent the normal processes of evolution [79]. Mathematical modeling has suggested that multiple-effect traits may be very common in ecological speciation [80].

It has also been proposed that the effect size of these traits is important, as that factor may determine directly whether the trait contributes to speciation [81]; furthermore, this effect size is dependent on the external environment and other modifiers [82]. We suggest using the more general term “dual traits” in describing traits that affect both ecological divergence and mating (or non-random gene flow) [39], regardless of the effect sizes of these traits. The effect sizes will vary for each empirical study, but the study of such traits will give us more insight into the genetic mechanisms and ecological factors underlying the speciation process. As the number of case studies begins to accumulate and we understand more about the mechanisms that underlie the evolution of such traits, they may not seem so “magic” anymore.

**Figure 1:**
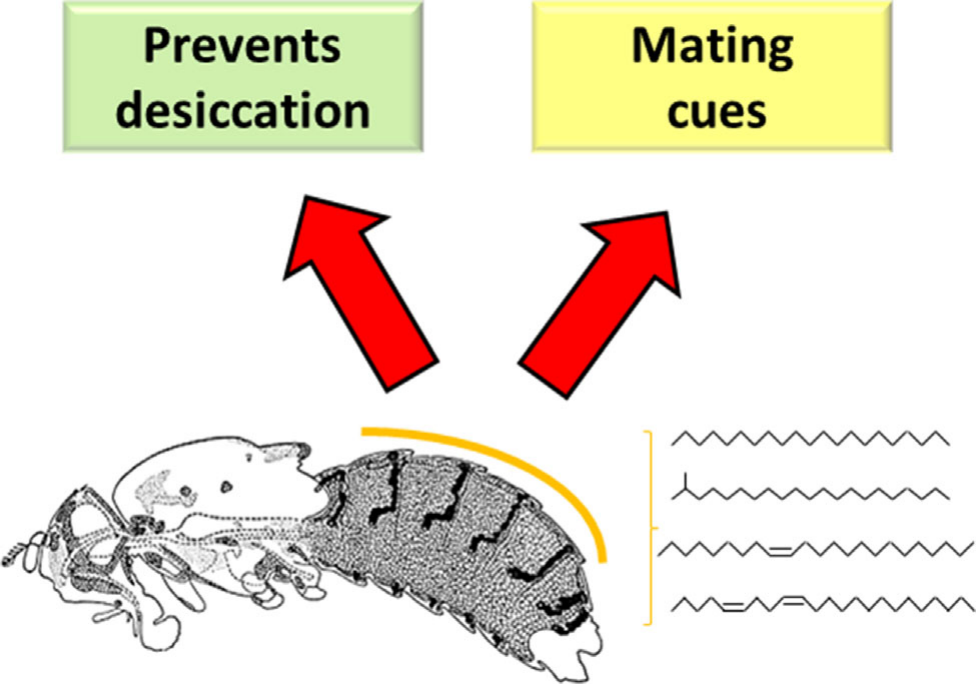
Cuticular hydrocarbons (CHCs) play two critical roles in insects. They form a waxy layer on the cuticles of insects to prevent desiccation due to cuticular water loss. Some of these CHCs have roles in mating cues. (Picture of oenocytes taken from [76]).

Insect cuticular hydrocarbons (CHCs) have been proposed to be potential dual traits [6]. Insect CHCs are long-chain hydrocarbons (mainly alkanes, alkenes, and branched alkanes) that are synthesized in specialized cells called oenocytes (Fig. 1), and subsequently transported to the cuticle of the insect. First discovered over 80 years ago as a layer of wax that controls water loss ([7,8] cited by [6]), hydrocarbons are believed to be the principal components of this layer, as their removal by organic solvents causes an increase in the rate of desiccation [9]. Insect CHCs have also been shown to have pheromonal activities [10], as well as roles in other forms of chemical communication [11], including mimicry [12], reproductive division of labor in social insects by signaling individual reproductive status [13,14], and courtship inhibition between closely related species [15]. However, despite the progress made in the identification of individual CHC or class of CHCs in chemical communication, until recently no studies had demonstrated that the same CHC or class of CHCs were also involved in ecological adaptation, and thus there was no causal evidence for CHCs functioning as dual traits (Box 1).

In this review, we suggest that the biology of insect CHCs may offer important insights into the process of ecological speciation for several reasons. First, insects are highly speciose, have a broad ecological distribution, and are adapted to many diverse habitats. Second, CHCs are chemically very diverse, and different insects produce different combinations of CHCs. Third, the mating systems of many insect species have been characterized in which CHCs play major roles. And finally, the biosynthetic pathway of insect CHCs is well characterized [16]. A number of the genes and enzymes that function in the pathway have been identified such that it is now possible to identify specific loci involved in the divergence of CHC composition and insect species. Here, we will discuss some of the recent evidence that has linked CHCs to ecological divergence and mate choice, and how the interaction between these two roles can lead to reproductive isolation. We suggest that insect CHCs provide good inroads to the study of dual traits and understanding the functional links between ecological adaptation and speciation.

## Insect CHCs have roles in both physiology and chemical communication

Maintaining water balance is critical for insect survival. Although there are multiple ways in which insects manage water loss [17–19], control of loss through the cuticle is a major route [18,20]. A water-proof layer composed of mixtures of straight-chain, methyl-branched, and unsaturated CHCs, with chain lengths ranging from approximately 21 to 50 carbons, provides a hydrophobic barrier against this loss [11,21]. Removal of this layer by physical methods, such as using solvents [9] or by genetic methods [22], renders the insect extremely sensitive to desiccation. The ability of this CHC layer to prevent desiccation depends on its composition, which in turn determines its melting temperature. In general, longer-chain CHCs have higher melting temperatures, while methyl-branched and unsaturated CHCs have lower melting temperatures [23].

It has been hypothesized that as insects adapt to new habitats or environments with different humidity levels, the composition of this waxy layer evolves to manage water balance. The observations that the desert drosophilid, *D. mojavensis*, is much more desiccation-resistant than other *Drosophila* species [24], and produces a higher than average proportion of longer-chain CHCs [25,26], is consistent with this proposal. Furthermore, in two independent laboratory selection experiments, *D. melanogaster* flies selected for desiccation resistance evolved longer-chain CHCs than control flies [27,28].

Chemical communication is also critical for insect lifestyles. CHCs facilitate communication by conveying various types of information. The most common role of CHCs is in mate recognition, where insects use CHCs as pheromones to attract potential mates [29]. CHCs also affect a wide variety of other behaviors including learning, aggregation, and dominance [30]. In social insects, CHCs convey information about nestmate recognition as well as functioning as fertility signals [31]. Some species exploit this nestmate recognition system by manipulating their CHCs during nest invasion to match the CHC of their host [12]. The identities of some of the CHCs that mediate chemical communication have been elucidated. For example, the natural CHC pheromone (Z)-9-tricosene (9-C23:1) has been shown to be produced by female houseflies (*Musca domestica*) to attract males [10]. In *Drosophila melanogaster*, 7-tricosene (7-C23:1) has been found to inhibit courtship and promote aggression between males [32]. And in the long horned beetle *Mallodon dasystomus*, two methylbranched CHCs, 2-methylhexacosane (2Me-C26), and 2-methyloctacosane (2Me-C28), are required for the full expression of mating behavior [33].

## A common biochemical pathway underlies the synthesis of diverse insect CHCs

Insect CHCs are exceptionally diverse. A survey of 78 ant species revealed a total of 187 distinct CHCs that occur in various unique combinations in each species, independent of their phylogenetic relationships [34]. In Drosophila, each species possesses a unique CHC blend, and in certain species such as *D. melanogaster*, this CHC blend is sexually dimorphic [26]. Variations in CHC blends in different populations of a single species have also been described [35,36].

Despite this diversity of CHC blends in insects, the main biosynthetic pathway for all CHCs is conserved [16]. CHCs are synthesized in the oenocytes from acetyl-CoA, which undergoes an elongation reaction to form a long-chained fatty acyl-CoA. This initial elongation reaction is catalyzed by either a microsomal fatty acid synthase – which catalyzes the elongation of branched fatty acyl-CoA which are the precursors of methylbranched alkanes (mbCHCs) – or a cytosolic fatty acid synthase – which catalyzes the elongation linear fatty acyl-CoA which are the precursors of linear alkanes (alkanes and alkenes). Elongases then further lengthen these fatty acyl-CoAs to specific lengths, and desaturases add double bonds during the chain elongation process [16]. The fatty acyl-CoAs are then reduced to aldehydes by fatty acid reductases, before a decarbonylation reaction mediated by a cytochrome P450, (the *CYP4G1* gene in *Drosophila*) converts the aldehydes to hydrocarbons [37] (Box 2). Importantly, oenocyte-specific knockdown of *Cyp4g1* expression results in the almost complete loss of all detectable CHCs [37]. This loss is accompanied by a substantial reduction in desiccation resistance, as well as decreases in female courtship and copulation behavior by control males.

**Box 2 Genes in the CHC synthesis pathway**

The CHC synthesis pathway is presumably co-opted from the fatty acid synthesis pathway present in most organisms. CHC synthesis takes place in the insect oenocytes, where a P450 decarbonylase converts some of the products produced from this pathway to long chained hydrocarbons, which are then transported to the cuticle of the insect (adapted from [16]). Plants utilize a very similar pathway in their production of cuticular waxes [83]. As all the CHCs in this pathway share a common biochemical pathway in the oenocytes, evolutionary changes in the genes in this pathway could lead to the diversification of CHC profiles between insect species. Certain classes of genes, such as desaturases and elongases, would be more likely to generate variations in CHC profiles between incipient species because of their enzymatic activities (described below). Although numerous members of each gene family have been identified in the insect genomes, it is important to note that since the CHC biosynthesis pathway takes place in the oenocytes [16,22], only the genes which are expressed in these cells are candidates for involvement in CHC synthesis. We describe a number of these genes in *Drosophila melanogaster*, where genetic studies have elucidated some of their functions.

**Function: Synthesize fatty acyl-CoA from acetyl-CoA and elongate chain**

**Number in *D. melanogaster* genome: 3**

Biochemical studies in houseflies and cockroaches showed that insects possess two different forms of fatty acid synthases (FAS) involved in the synthesis of CHCs, a cytosolic FAS involved in the synthesis and elongation of non-branched CHCs (alkanes and alkenes) and a microsomal FAS, which is specific for the synthesis of and elongation of methyl-branched CHCs (mbCHCs) [84,85]. The annotated *D. melanogaster* genome contains three FASs [86], two of which are expressed in the oenocytes. A probable candidate for the microsomal FAS is CG3524 (mFAS). RNAi knockdown of CG3524 (mFAS) in the oenocytes eliminates the production of mbCHCs in two different Drosophila species. Evolutionary changes in FAS should not be very common, as the inactivation of FAS would eliminate a whole class of CHCs. One example, however, is in *D. birchii*, which has almost no mbCHCs, where *cis*-regulatory changes in CG3524 (mFAS) resulted in the loss of gene expression in oenocytes.

**Function: Elongate fatty acyl-CoA chain**

**Number in *D. melanogaster* genome: 19**

Elongases elongate the fatty-acyl coA chain by two carbons after the chain is synthesized by FAS [87]. Each elongase has sequence-specific enzymatic activity, which contributes to the diversity of fatty acids produced [88] and, therefore, to CHCs of different lengths. There are 19 fatty acid elongases in the *D. melanogaster* genome [89], but it is unlikely that all of these elongases are expressed in the oenocytes. Elongases such as Elo68 and bond, are expressed in the male reproductive system [90,91]. The only elongase that has been identified in *D. melanogaster* with a specific function in CHC biosynthesis is EloF, which is responsible for the production of 7,11-HD (C27:2) and 7,11-ND (C29:2) in female *D. melanogaster* [89]. Elongases are candidate genes for investigating CHC variations within or between populations differing in CHC chain-lengths.

**Function: Adding double bonds during synthesis and elongation of the fatty acyl-CoA chain**

**Number in *D. melanogaster* genome: 9**

During chain elongation, desaturases add double-bonds to the fatty acyl-CoA chain. The activities of desaturases are also gene-specific, which leads to the diversity of unsaturated CHCs found on insect cuticles [59,92]. There are nine desaturases in the *D. melanogaster* genome [93], and three of them are involved in the synthesis of CHCs. *desat1* and *desat2* add a single double bond at either the seventh carbon or fifth carbon to the fatty acyl-CoA chain during chain elongation, respectively [92]. *desatF* adds a second double bond to the fatty acyl-CoA chain and is responsible for the production of dienes in *D. melanogaster* females [94]. In the Drosophila genus, *desatF* has undergone many evolutionary transitions, such as gene loss, loss of oenocyte expression and the evolution of sexually dimorphic expression [95], which leads to the extensive transitions in the production of dienes. Because unsaturated compounds often play a role in chemical communication, and the number of desaturases varies among closely related species, desaturases are good candidates for investigations into reproductive isolation due to changes in chemical signaling [93,96].

**Function: Convert fatty acyl-CoAs to aldehydes**

**Number in *D. melanogaster* genome: 17**

Fatty acid reductases convert the acyl-CoA side chain of the long chain fatty acyl-CoA after elongation to an aldehyde [16]. There are 17 fatty acyl-CoA reductases in the *D. melanogaster* genome [86] but there have been no studies thus far as to whether evolutionary changes in reductases cause any changes to CHC biosynthesis in oenocytes within or between species.

**Function: Convert aldehydes to hydrocarbons**

**Number in *D. melanogaster* genome: 85**

Cytochrome P450s are heme-containing enzymes capable of performing a wide variety of chemical reactions and are involved in diverse biological processes such as hormone synthesis and resistance to xenobiotics [97]. After fatty acid reductases convert long chain fatty-acyl coAs to aldehydes, a cytochrome P450 converts these aldehydes to hydrocarbons in a decarbonylation reaction [98]. Genetic and biochemical experiments identified the P450 responsible for this reaction as *Cyp4g1* [37], which is the only P450 detected in Drosophila oenocytes so far. RNAi knockdown of *Cyp4g1* in *D. melanogaster* oenocytes resulted in the elimination of almost all CHCs. It is unlikely that evolutionary changes in this P450 enzyme would be responsible for CHCs differences between species, because any changes to *Cyp4g1* would affect almost all CHCs in the insect.

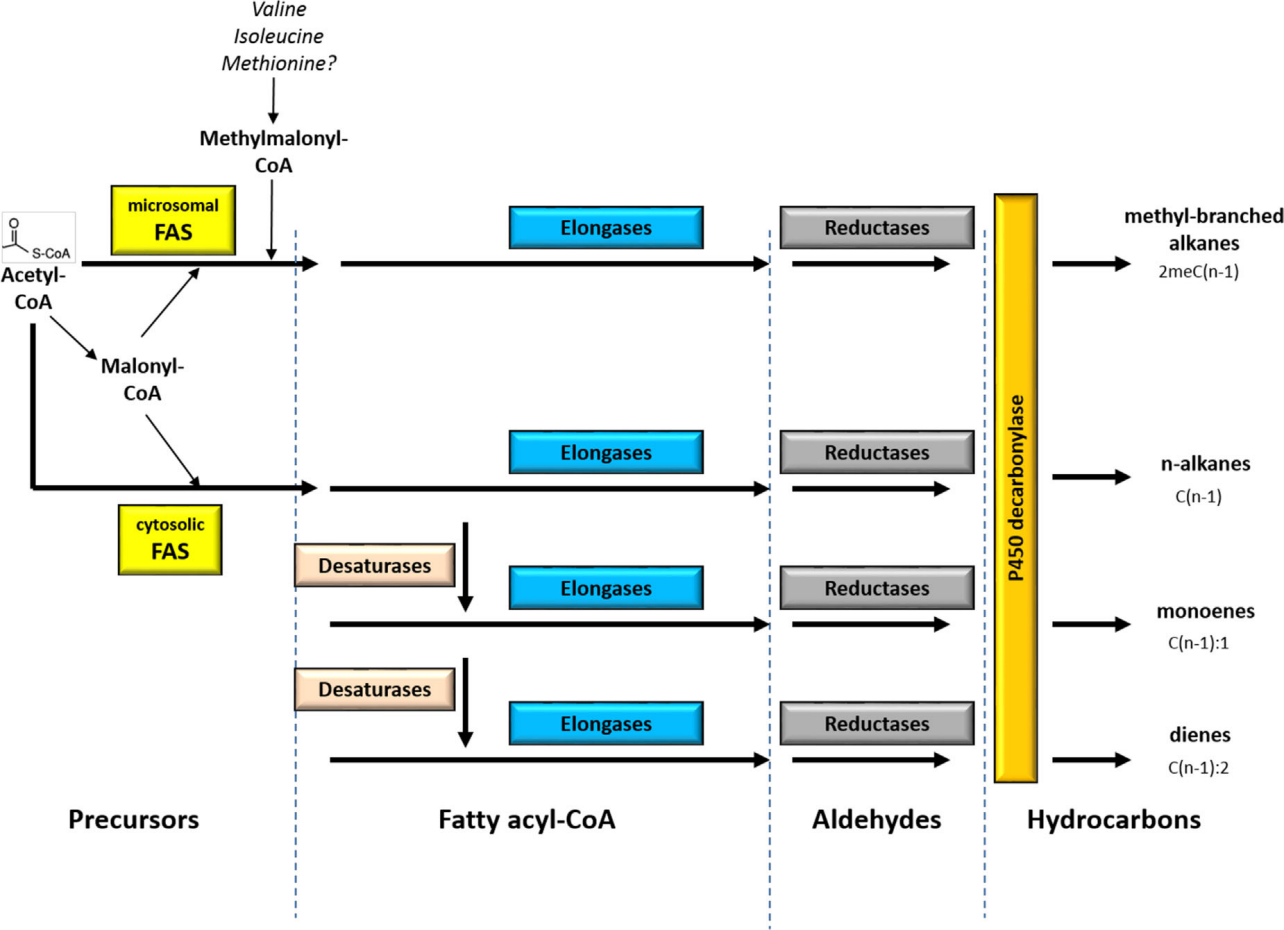

## Insect CHCs are potential dual traits

The demonstration that CHCs as a group have roles in both desiccation resistance and chemical communication raises the possibility that individual CHCs, or certain classes of CHCs, might function in both processes. If true, then CHCs could operate as dual traits in instances where ecological adaptation affects the production of CHCs that are also involved in mating success.

**Figure 2:**
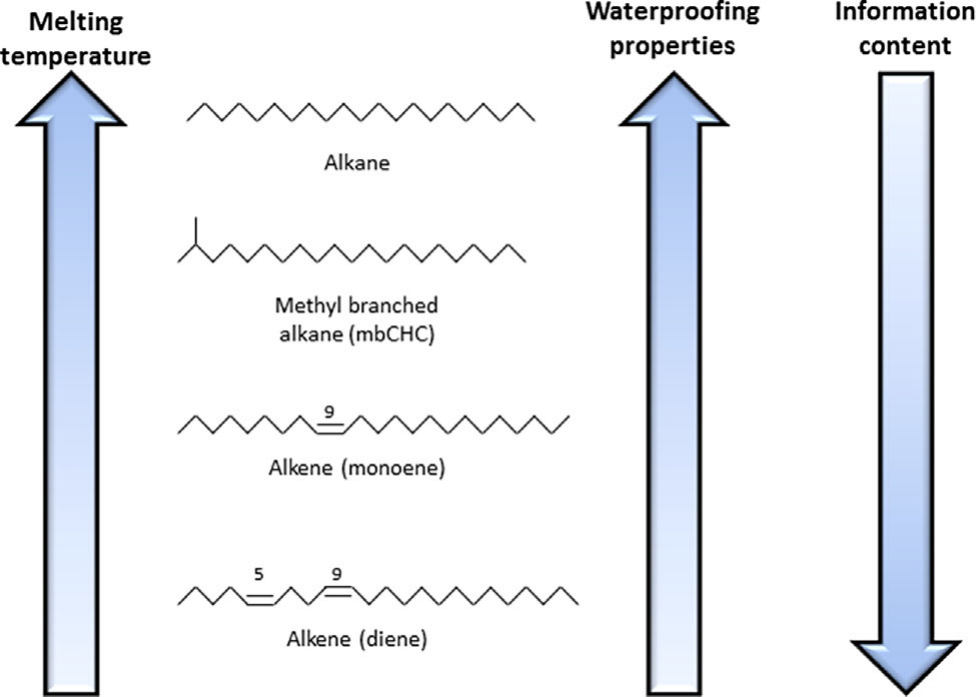
Melting temperatures of CHCs are directly correlated with waterproofing properties but inversely correlated with information content.

**Figure 3:**
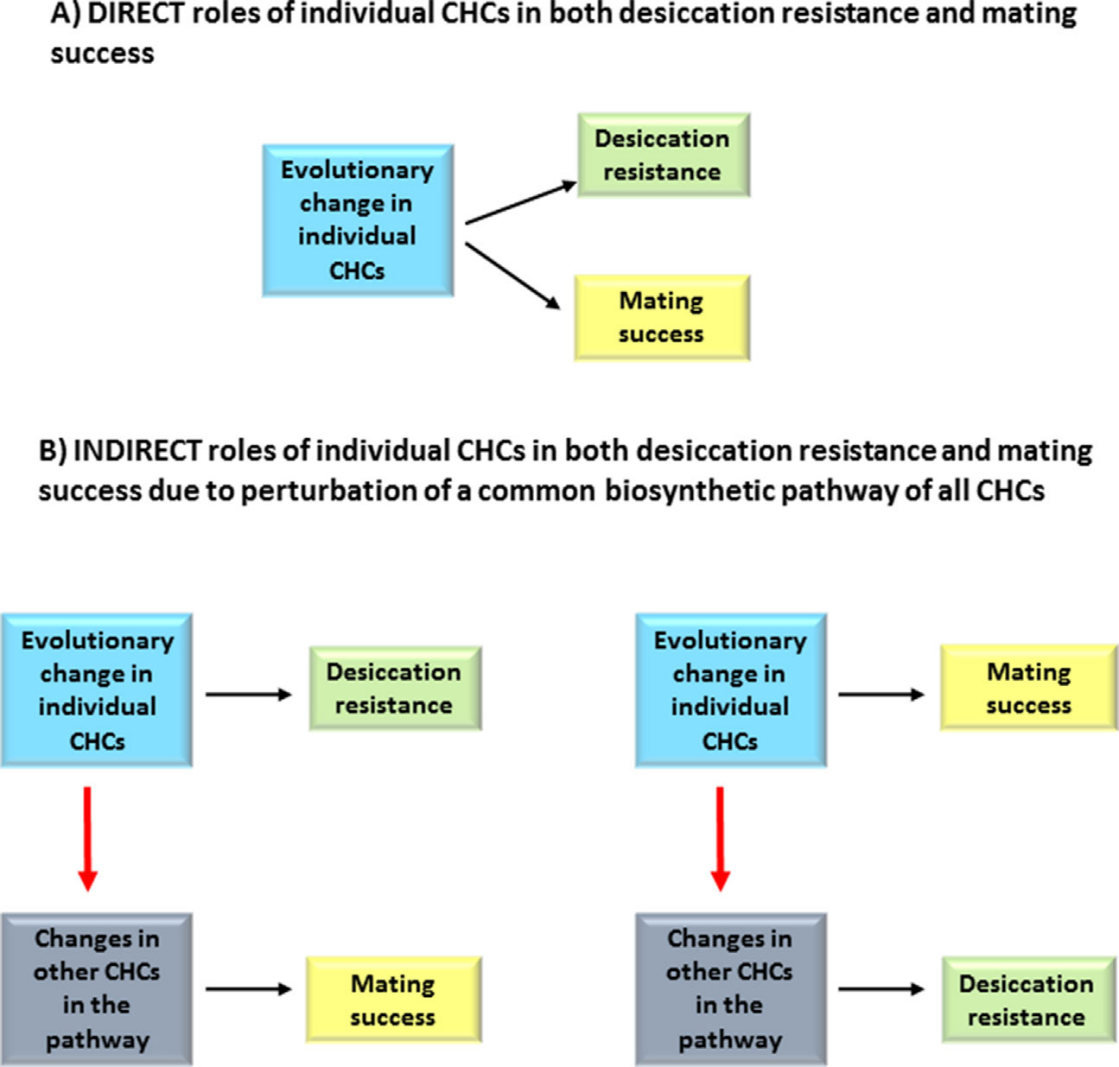
Two models for how CHCs can affect both desiccation resistance and mating success. A: The direct model. Evolutionary changes in CHCs may affect both desiccation resistance and mating success when these CHCs play roles in both processes. B: The indirect model. Evolutionary changes in CHCs that affect either desiccation resistance or mating success may affect the other process by altering the production of other CHCs in the pathway.

However, there have been few attempts to study CHCs as potential dual traits. One reason for this neglect may be that it was long thought that the CHCs involved in waterproofing are long-chained saturated hydrocarbons (*n*-alkanes) with high melting points but not much structural diversity while CHCs involved in chemical communications are more diverse and have the potential for high information content, but their low melting points reduces their water proofing potential (Fig. 2). However, there is evidence that compounds with intermediate melting temperatures and volatility, i.e. the alkenes and methyl-branches alkanes, could directly affect both desiccation resistance and chemical communication (Fig. 3A).

Moreover, because insect CHCs are synthesized from a common pathway that utilizes Acetyl-CoA as a substrate for chain elongation, it is possible that changes in the production of one CHC, or class of CHCs, could also affect the production of other CHCs that function in chemical communication or desiccation resistance. In this scenario, the changes in one CHC or class of CHCs would have an indirect effect on another CHC or class of CHCs (Fig. 3B), but would exert the same biological effect as a dual trait. We discuss examples of these two different scenarios in which CHCs could behave as dual traits.

### Direct dual roles of individual insect CHCs

The intermediate melting temperatures of monoenes and methyl-branched alkanes endows them with the potential of serving roles in both reducing water loss and mediating chemical communication. One potential example is in *D. melanogaster*, where male flies that are found in warmer regions nearer to the equator have higher levels of the longer-chain monoene 7-pentacosene (7-C25:1, or “7-P”) as their main hydrocarbon, compared to flies found in cooler regions further from the equator, which have higher levels of the shorter-chain 7-tricosene (7-C23:1, or “7-T”) as their main CHC [35]. Flies with higher levels of the longer 7-P have greater desiccation resistance [36]. In addition, a laboratory selection experiment found that flies with higher levels of 7-P have a selection advantage at higher temperature. The selected flies with higher 7-P levels also exhibited partial sexual isolation from control strains, suggesting that environmental factors could lead to CHC evolution and decreased mating success [38]. However, while this study showed that individual CHCs, such as 7-T or 7-P, could be correlated with both desiccation resistance and mating behavior, it does not provide adequate evidence of their direct involvement in both processes, because other factors that affect desiccation resistance could not be controlled for in the experiments.

We have recently demonstrated a direct role for one class of CHCs in both desiccation resistance and mating success in *Drosophila* [39]. The Australian fruitflies *Drosophila serrata* and *Drosophila birchii* are a pair of closely-related species with overlapping ranges on the east coast of Australia [40]. *D. serrata* is a habitat generalist found outside of and on the fringes of the rainforest while *D. birchii* is a habitat specialist found exclusively in the humid rainforest. *D. serrata* is significantly more desiccation resistant than *D. birchii* [41]. These two species exhibit strong premating isolation [42,43] in which chemical cues are shown to be important for mate recognition [43,44].

The CHC blends of the two species differ greatly: *D. serrata* has relatively high amounts of methyl-branched CHCs (mbCHCs) while *D. birchii* produces only trace amounts [45]. The methyl-branched CHCs (mbCHCs) have melting points above ambient temperature and could play a role in preventing water loss [23]. They have also been implicated in influencing mating success in both *D. serrata* [45–47] and other insects [14,33]. One potential explanation then for the divergence of these two species is that mbCHCs serve as a dual trait. In this scenario, ecological adaptation to the humid rainforest may have led to the loss of mbCHCs in *D. birchii* and reproductive isolation from *D. serrata*.

To test this hypothesis, we specifically manipulated levels of mbCHCs. We identified a gene encoding a mbCHC-specific fatty acid synthase, *mFAS* (*CG3524*), as the gene responsible for producing mbCHCs in the oenocytes [39]. We found that RNAi-mediated knockdown of *mFAS* expression in *D. serrata* led to a striking reduction in both desiccation resistance and male mating success. Furthermore, application of pure synthetic mbCHCs to *mFAS*-RNAi *D. serrata* flies was able to partially restore desiccation resistance, and the application of one specific mbCHC, 2Me-C26, was able to increase mating success in *mFAS*-RNAi as well as wild-type flies [39]. Together, these results demonstrate that mbCHCs function directly as a dual trait in *D. serrata*. We also found that *mFAS* expression had been lost in the rainforest-adapted *D. birchii*. Therefore, evolutionary changes in the expression of *mFAS* and mbCHCs may have contributed to pre-mating isolation between *D. serrata* and *D. birchii*.

### Indirect dual roles of insect CHCs

In principle, evolutionary changes in the genes that govern the production of individual CHCs might also indirectly affect other CHCs produced by an insect. This has been demonstrated in *Drosophila*, where genetic manipulation of fatty acid synthesis gene expression in oenocytes led to an increase in one class of CHCs and reduction in another class of CHCs. For example, knockdown of *mFAS* expression and mbCHC synthesis in *D. serrata* also led to a small increase in diene levels [39]. Similarly, overexpression of the *desat1* desaturase gene in *D. melanogaster* oenocytes led to an increase in unsaturated CHCs such as monoenes and dienes and a decrease in alkanes, and the opposite was observed when *desat1* expression was knocked down by RNAi [48]. Knockdown of another desaturase gene, *desatF*, in oenocytes, also led to a decrease in dienes but an increase in monoenes [48].

Changes in the expression of one class of CHCs can, therefore, lead indirectly to the evolution of another class of CHCs (Fig. 3B), which in turn may influence a different process. In such situations, natural and sexual selection on the production of certain CHCs may conflict. One potential example of this effect is in female houseflies, *Musca domestica*, which use unsaturated alkenes such as (Z)-9-tricosene (9-C23:1) as a sex pheromone. Montooth and Gibbs showed that immature houseflies have lower amounts of these alkenes but have higher amounts of mbCHCs than mature houseflies. While the CHC blend of the mature houseflies are more attractive, the younger houseflies exhibit lower water loss [49]. These observations suggest some potential conflicts and tradeoffs between the composition of CHCs on the cuticle that is optimal for limiting water loss and the composition that optimally mediates sexual attractiveness, which may be an example of the classic handicap principle [50,51].

## CHC composition can evolve as a consequence of other ecological adaptations

While we have focused on desiccation resistance as a major ecological factor in CHC evolution, other ecological variables may also cause insect CHC blends to evolve. Adaptation to these factors and the concomitant divergence in CHC profiles may also contribute to reproductive isolation.

Several instances of dietary influences on CHCs have been documented in *Drosophila*. For example, in *D. mojavensis*, variations in CHC blends between different populations are thought to be caused by feeding on different host plants [52,53]. In *D. serrata* as well, adaptation to different diets generated different CHC blends, and led to differential mating success and pre-mating isolation [54]. In addition, laboratory experiments with *D. melanogaster* showed that providing yeast versus sugar drove female CHC profiles in opposite directions, although there was no observable effect on female attractiveness [55]. Dietary effects on CHCs have also been shown for other insect species such as beetles [56] and ants [57]. One reason that CHC blends evolve in conjunction with adaptation to different food sources is that the production of certain classes of CHCs such as the mbCHCs requires certain amino acids such as leucine, isoleucine, and valine (see methylmalonyl CoA precursors in Fig. 2). Different food sources may provide different quantities of amino acids, leading to different outputs of the CHC pathway in the production of fatty acids and CHCs.

The fatty acid synthesis pathway that produces CHCs in oenocytes is also active in other cells for the production of fatty acids and other lipids such as membrane phospholipids. Alterations to the desaturation levels of these membrane phospholipids have been suggested to affect cold tolerance because these lipids help to maintain membrane fluidity at low temperatures [58]. Therefore, adaptation to cold could also cause changes in fatty acid synthesis, leading to changes in CHC blends and potential reproductive isolation. One case that has been extensively studied concerns the divergence in the activity of the *desat2 (ds2)* desaturase gene in different populations. *D. melanogaster* females from African populations (Z lines) carrying the *ds2^Z^* allele produce 5,9-heptacosadiene (5,9-HD), while cosmopolitan females (M lines) carrying the *ds2^M^* allele produce 7,11-heptacosadiene (7,11-HD), due to the inactivation of the *ds2* locus [59]. M lines are more cold tolerant than Z lines, and there is pre-mating isolation between African and cosmopolitan *D. melanogaster* flies [60]. Greenberg et al. hypothesized that the pre-mating isolation caused by the two different alleles of *ds2* was a result of ecological adaptation to cold tolerance [61]. To test this hypothesis, precise gene targeting was used [62] to replace the *ds2^M^* allele with the *ds2^Z^* allele in M lines. Greenberg et al. reported that the M lines with *ds2^Z^* allele produce 5,9-HD and became susceptible to cold as compared to control lines [61], and subsequent experiments showed that the transgenic flies recapitulate the pre-mating isolation between the wild type African and cosmopolitan flies [63]. Although there has been considerable controversy about this study [63–65], it was the first successful effort to manipulate a single CHC biosynthetic pathway locus and advanced the general hypothesis that ecological adaptation could lead to evolution of CHC blends and reproductive isolation because of the pleiotropic effects of the genes involved in CHC synthesis.

In social insects, the evolution of CHCs may depend on contrasting selection pressures that act on particular compounds. In the trap-jaw ant *Odontomachus brunneus*, nestmate signatures and fertility signals both involve CHCs. While nestmate signatures are a collective mixture of CHCs, fertility signals are often a single compound or a subset of compounds within that blend [66]. While CHC profiles are highly variable between different populations of *O. brunneus*, one of these CHCs, (Z)-9-nonacosene (9-C29:1) is used and conserved as a fertility signal across populations [31], although the levels of (Z)-9-nonacosene differ between populations. These observations suggest that the evolution of (Z)-9-nonacosene is dependent on both natural selection that produces divergent nestmate recognition CHC profiles, as well as sexual selection for a conserved fertility signal.

## Conclusions and outlook

We have discussed merely a handful of case studies that have implicated CHCs in insect adaptation, mating success, or speciation. But this modest sample of well-studied species may be just a glimpse into a more widespread role for CHCs in the diversification of insects. CHCs are both nearly ubiquitous and remarkably diverse compounds, and insects are the most speciose group of animals on the planet [67]: one that has adapted to a great variety of habitats. Could there be a causal relationship between this chemical and phylogenetic diversity?

Until recently, the major experimental barrier to understanding the possible general roles of CHCs was that the sorts of genetic resources and tools necessary for elucidating CHC regulation and specifically manipulating CHC levels were available for only a single insect species, *D. melanogaster*. However, although *D. melanogaster* has a widespread distribution, it has been suggested that its ecology made it an unsuitable model to study the causes of speciation in nature [68]. The ability to test the general role of CHCs as drivers of ecological adaptation and speciation in insects has now become feasible as a result of several recent advances. First, insect genomes are amenable to sequencing quickly and inexpensively because of the advent of very high-throughput technologies [69]. Second, techniques for gene knockdown, gene overexpression, and genome editing are now widely applicable to non-model species [70,71]. Third, the decreasing costs of sequencing technologies also make it feasible to identify candidate genes underlying CHC divergence between closely related insect species using population genomics [72]. Together with new methods for the isolation and synthesis of CHCs [73,74], it is now possible to experimentally test hypotheses about the roles of CHCs in nature.

The production of CHCs, however, is only one facet of the divergence in chemical communication. One aspect of CHC biology that is significantly understudied is the perception of these compounds. As CHC blends diverge and new mate recognition systems evolve, it is expected that the receptors and responses to CHCs will also diverge. In *D. melanogaster*, many of the receptors for CHCs have been characterized [15,75], but how they co-evolve with the divergence of CHCs within populations is not known. This is a very important area of research that is necessary to link the evolution of chemical communication to behavior and reproductive isolation. One potential starting point for investigations may be to examine partially reproductively isolated populations of a single species that exhibit CHC polymorphisms and assortative mating. Classical genetic mapping coupled with population genomics could identify candidate loci that are involved in the perception of CHCs.

As a young man, Darwin was fascinated with his beetle collection because of their diversity. The study of this layer of wax found on the surface of insect cuticles may provide some fresh insights into Darwin's idea of how ecological adaptation can lead to the origin of new species.

## Abbreviations

CHCs: cuticular hydrocarbons
mbCHCs: methyl-branched CHCs
